# From paediatrics to geriatrics: a life course perspective on the MRC National Survey of Health and Development

**DOI:** 10.1007/s10654-016-0214-y

**Published:** 2016-12-21

**Authors:** Diana Kuh

**Affiliations:** MRC Unit for Lifelong Health and Ageing at UCL, London, UK

## Introduction

For over 40 years I have enjoyed working collaboratively on research projects to increase understanding of population health with the aim of ultimately improving quality of life. For almost 30 years I have been part of the study team responsible for the MRC National Survey of Health and Development (NSHD), the oldest of the British birth cohort studies; and for the last 10 years have had the privilege of being the NSHD director. Such a long-term study depends on a committed and scientifically productive study team which maintains study member engagement and attracts expert scientific collaborators. For the last 20 years, I have collaborated with Professor Yoav Ben-Shlomo and others to develop the field of life course epidemiology, the study of the long term effects of social and biological exposures and experiences across life on later life health. So at the outset, I acknowledge that this personal opinion piece which I was invited to submit has implicitly and explicitly been influenced by many colleagues. It also should be read in conjunction with an accompanying article in this issue on the recent 24th follow-up of the NSHD at the age of 70 years (Kuh et al., this issue) and a recent updated review of life course epidemiology [1].

## Background

I first recognised the degree of heterogeneity in the health of older people and pondered its origins in 1974–1975 when delivering meals on wheels to a group of older people in the US state of Vermont as part of the Federal Council on Aging programme. What lifetime experiences had enabled one 85 year old to remain sufficiently robust to tend his few acres while another, at least a decade younger but already frail, lived in squalid surroundings in a run-down local boarding house? On returning to the UK, my interest in life course ideas—how experiences earlier in life can shape health and other life chances—was sparked by two informative experiences in my early career. During my first UK position as an operational research scientist for the Department of Health in an outpost at Exeter University (1975–1981), I helped to build computer models to inform the resource allocation of health and social care expenditure to different client or patient groups. The lack of discussion about the quality of care and its impact on people’s lives was frustrating. One of my first research tasks was to interview the nursing staff of an old hospital for adults with mental and behavioural impairments about the abilities of the inpatients on each ward, and whether they were seen as suitable (or not) for community care. There were photographs of the patients before admission in their files, many as young people, and hearing about their reasons for admission and parts of their life stories, gave me a glimpse of the lifelong impact on individuals of their environment. So I was attracted by a job offer from one of the first paediatricians in the NHS, Professor Frederic Brimblecombe, an inspirational clinician who knew the value of integrated and high quality holistic care services for children with disabilities [2]. He asked me and a paediatric registrar to run a research project (1982–1987) that involved interviewing 400 young people with physical and mental disabilities about their unmet health and social needs after they left paediatric care, and then making a case for changes to local services to meet those needs. Hearing about the factors that had shaped these young people’s lives, their lack of opportunities, the impact of the social and care environment and how public services could make a difference left a deep impression.

By chance, a factor of considerable importance in shaping lives [3], I met Professor Michael Wadsworth, then the NSHD director, who told me how Dr James Douglas, a physician with an interest in social medicine, had started this birth cohort study in March 1946, following up 5362 babies born in one week that month in England, Scotland and Wales. Wadsworth and others have captured the first 40 years of NSHD history elsewhere [4–6]. Some of the key findings relate to: the social inequalities in infant health and survival, and childhood educational opportunity and attainment, even of children with high measured cognitive ability; the power of parental interest for the child’s education independently of school and family background; the increased risk of adolescent behaviour problems in those who experienced long or repeated hospital admissions in early life, and the disruptive effects for the child of parental divorce and separation.

I took up a post in the NSHD team in 1987, soon after Wadsworth had moved the team to UCL. Wadsworth had kept this birth cohort study alive against the odds in the late 1970s and early 1980s after Douglas retired [7]. The MRC, who had started funding NSHD in 1962, agreed in 1981 to give the study a 5-year reprieve to assess its benefits in understanding the development of mental and physical ill-health and its change in young adults, including continuous measures of blood pressure and respiratory function. With new data collected at 36 years, Wadsworth showed that babies of low birthweight had higher adult blood pressure [8]. On publication of this paper in the BMJ, he received a phone call from the late Professor David Barker to say how he had just beaten him in the publication of similar data. This was to mark the end of a period of relative famine in cohort investment in the UK. The late 1980s and 1990s was an opportune time to arrive in the world of cohort studies and chronic disease epidemiology. Barker’s imaginative research using historical cohorts, and his fetal origins hypothesis attracted a lot of attention, stimulated a new research agenda, and was a catalyst in the revival of a life course perspective in epidemiology. Visiting Barker to discuss a collaborative paper was a high point of my first year on the NSHD team; this paper [9] remains one of my most highly cited, and the discussion we had that day inspired me to focus my PhD thesis on testing the Barker hypothesis in NSHD. The proliferation of papers and books from Barker and his team stimulated a group of young epidemiologists, many of whom were critical of Barker’s methods and questioned his interpretation of the data, to: review the literature on pre-adult risk factors associated with cardiovascular and respiratory diseases, diabetes and cancers; explore the social and biological pathways between early life and adult disease; and consider the lessons to be learnt from time trends, geography and migration, and socioeconomic differentials. With Yoav Ben-Shlomo, I took the opportunity to turn these reviews into the first textbook on a life course approach to chronic disease epidemiology [10]. The emergence of the field of life course epidemiology which this book fostered had, for me, a synergistic relationship with the direction of NSHD research over the subsequent decades, each influencing the other.

## The NSHD in its 4th, 5th and 6th decades

This new scientific agenda on the early origins of adult disease required investment in birth cohorts and revitalised historical cohort studies as they provided the empirical evidence to generate and test emerging developmental and life course hypotheses. Between 1982 (when NSHD participants were age 36) and 2006 (at age 60), Wadsworth directed the NSHD with the aim of studying pathways to physical and cognitive ageing with a particular focus on the influence of early life factors. Home visits by research nurses, first initiated at the 36 years follow-up, were used again at ages 43 and 53 years, the core set of functional assessments was expanded and the first blood sample collected. In addition, we initiated a study of women’s midlife health, by sending regular postal questionnaires from 47 to 57 years to women study members to capture the menopause transition and changes in their midlife health. High participation rates of over 80% were maintained at all these follow-ups, helped by birthday cards and regular feedback to study members, and by responding personally to those with queries and those who shared additional life experiences.

In brief, we used these data to demonstrate a large range of associations between the early environment, physical, cognitive and emotional development and functional ageing and age-related diseases in midlife, focusing on cardiovascular, musculoskeletal, respiratory and mental health, cognitive function and women’s reproductive health. This research is summarised elsewhere [11], and references to all publications are on the study website (www.nshd.mrc.ac.uk). The NSHD research I led during this period explored the developmental and early environmental origins of musculoskeletal and reproductive ageing, and of premature mortality. In brief, spurred on by my prior interest in disability, and my collaborators’ interests in musculoskeletal function, baseline measures of grip strength, standing balance and chair rise time were introduced at the home visit at 53 years [12]. We were able to show how the early social environment, patterns of childhood growth and neurodevelopmental measures were related to these functional measures 40–50 years later, over and above the effects of adult health and lifestyle [13–17]. In terms of women’s health, we found that developmental factors, such as being breast fed, infant weight gain, and higher childhood cognitive ability were associated with a later onset of menopause whereas adverse early socioeconomic circumstances (father’s social class, parental divorce) were associated with an earlier onset, independent of adult risk factors of nulliparity and smoking [18–22]. We also published a series of papers about lifetime factors associated with premature mortality, focusing initially on early factors, such as father’s social class, parental education and childhood cognitive ability [23–28]. By age 53, NSHD already had repeat measures of adult cardiovascular and cognitive function so studies of functional change were possible by others in the team (for example [29–31])

The key public health message from the team’s publications was that childhood mattered for adult health, and that investment in the health and early environment of children would lay the foundations for adult health. At that time, evidence of the long-term impact on health and life chances of early interventions in the US was being published [32].

During this period, parallel with the NSHD research, I worked with others to develop conceptual frameworks, models and methods for life course epidemiology and apply them to a growing number of health outcomes, modelling trajectories of risk (e.g. growth trajectories) and increasingly studying functional trajectories or preclinical traits that enabled the study of lifelong health before chronic diseases were manifest [33–35].

## The NSHD in its 7th decade

In 2007, I was appointed Director of the NSHD, and with a remit to establish the MRC Unit of Lifelong Health in Ageing and to transform the NSHD into a world class, interdisciplinary study of ageing. Population ageing was firmly on the political [36], social and research agendas in the UK, as it was in other countries. There was and still is a great pressure in the wider society to understand better the social and biomedical factors that either accelerate or slow down the rate of ageing. Within life course epidemiology, there was also a growing focus on ageing, the progressive decline in function, as this was a natural extension of investigating functional trajectories. There was, and still is, a lack of consensus on the definition of healthy ageing [37], so we applied a pragmatic definition to help frame the Unit’s thinking [38]. Healthy biological ageing was represented by survival to old age, delay in the onset of chronic disease and optimal functioning for the maximum period of time at the individual level (which we termed physical and cognitive capability), and the underlying body systems on which capability depended. Social and psychological wellbeing, while important for healthy ageing, did not necessarily decline with age, and was studied separately, facilitating studies of bidirectional relationships, concordance and discordance between healthy biological ageing and wellbeing.

During these 10 years, the team has undertaken two major data collections, authored 500 publications, increased the international and media profile of NSHD, transformed the original card based metadata records into a series of 21st century web-based data discovery, sharing and governance tools, and instigated transparent data sharing policies and procedures to support the huge increase in data sharing. We have become an international centre for life course epidemiology, inter-cohort studies, and training and capacity building.

### The NSHD follow-up at 60–64 years

Our pragmatic definition of healthy ageing informed a pioneering new model of data collection for the NSHD. Between ages 60 and 64, we asked study members who live in England, Scotland and Wales, to attend one of six clinical research facilities or to have a research nurse visit them at home [39]. Working with expert collaborators, clinic attendance allowed us to use imaging techniques to obtain information on the structure and function of the heart and blood vessels, and on body composition (using DXA and pQCT), collect more biological samples, and repeat our core measures of physical and cognitive capability and mental health. Study members responded positively to this request and we achieved an 84% overall participation rate [40].

With these data, we showed, for example, the effects of childhood and adult risk factors on bone, lean and fat mass [41–48], on cardiac and vascular structure and function [49–51], and on functional change [52–54]. Variation in midlife physical and cognitive capability was meaningful in terms of later mortality risk [55, 56]. These data also allowed a more detailed assessment of fifteen common clinical disorders where there is UK consensus about monitoring and treatment by a GP or other clinician [57]. On average, participants had two or more clinical disorders as they reached retirement age, with less than one in six having none; and we continue to show the early life origins of many of these disorders [58–60], and their underlying risk factors [61, 62].

This period also saw a great increase in cross cohort studies, some initiated by the team and others to which we contributed. Under the *Healthy Ageing Across the Life Course* (HALCyon) collaborative programme, we brought together NSHD with eight other UK cohort studies to investigate the lifetime determinants of healthy ageing. Here we undertook systematic reviews, meta-analyses and cross cohort studies, complemented by in depth studies of single cohorts, to gather evidence about the extent to which early growth and later body size, childhood cognitive ability and the early environment, and aspects of the underlying biology (cortisol, telomeres and genetics) were associated with one or more of our measures of physical and cognitive capability or psychological and social wellbeing.

We brought the results of over fifty HALCyon publications (see www.halycon.ac.uk), and a review of the literature, including the team’s own research, together in a book on ‘A life course approach to healthy ageing’ [63]. In brief, we found that healthy biological ageing was affected by the same type of lifetime protective factors that reduced the risk of chronic disease: less socioeconomic adversity, no evidence of poor growth and development, maintenance of normal weight across adult life, good prior health and positive health behaviours. In contrast, wellbeing was less affected by lifetime socioeconomic circumstances and more affected by healthy relationships in childhood and adult life. One of our conclusions was that there should be more monitoring of risks and functional decline longitudinally, at least from midlife, to identify groups at risk of accelerated ageing who could benefit from timely interventions that could slow down ageing and promote physiological resilience, or promote social and psychological resilience in the face of accelerated functional ageing.

Cross cohort research was also expanded by setting up the NSHD DNA resource and samples bank in 2008 (under the careful leadership of Dr Andrew Wong). This enabled the use of NSHD data in many more consortia investigating the genetics of common diseases or traits and in Mendelian Randomisation studies of the environmental determinants of disease. We led or played a greater role in studies that showed how genetic associations with body composition or other health-related characteristics varied across life, acted on the tempo of growth in childhood or were associated with susceptibility to adult obesity [64–67]. Additional funding and expert collaborators have also enabled the first NSHD studies in epigenetics; and recent investments in ‘omic capabilities are currently facilitating new epigenetic and metabolomic studies of functional ageing.

### The NSHD follow-up at 68–69 years

The most recent data collection (a postal questionnaire in 2014 followed by a research nurse home visit in 2015–2016) took place at 68–69 years and high participation rates were maintained (Kuh et al. this issue). The first aim of this collection was to repeat our core measures of physical and cognitive capability in order to: (1) to look back and see whether relationships with earlier life factors were persistent, and which lifetime factors were associated with functional change; (2) to look forward to see whether midlife capability and any subsequent change were related to morbidity, disability and quality of life; and (3) whether they related to ageing at the body system, cellular and molecular levels.

The second aim of the data collection was to capture morbidity and multi-morbidity in more detail than before in order to: (1) to investigate their relationships with age-related disease, reduced physical and cognitive capability, and earlier life social and biomedical factors; and (2) to show how all these aspects of health jointly impaired wellbeing, quality of life and risk autonomy and independence at older ages. At this visit, we repeated our core mental health and wellbeing measures, introduced standardised scales to capture chronic pain [68], fatigability, sleep quality, incontinence and mental status, and collected more detail than before on functional limitations, activities of daily living, instrumental activities of daily living, the use of health and social services, and social participation.

These new data are just becoming available and many studies that extend the team’s previous research are ongoing. For example, we have already found that adding repeat measures of fluid cognition at age 69 to our three previous adult measures since age 43 are changing the shape of the cognitive trajectories and identifying certain groups with accelerated decline. We are developing approaches to study psychological and social resilience in older people [69, 70], for example those with high wellbeing despite functional decline. We are also developing approaches to study lifetime pathways to multi-morbidity, preliminary analyses having shown that over one in five now have three or more doctor diagnosed conditions (out of a possible 19), and that 29% women and 15% of men report three or more common conditions (chronic pain, fatigability, poor sleep quality, incontinence and falls).

### The neuroscience clinical sub-study 69–73 years

Having successfully brought study members into clinics to image the cardiovascular and musculoskeletal systems at 60–64 years, we are now collaborating with colleagues at the Institute of Neurology on an ambitious neuroscience sub-study in NSHD, called Insight 46 which has secured additional funding. It requires participants from across mainland Britain to travel and stay overnight in London, consent to a PET scan requiring the use of a tracer, and to a battery of neuropsychological tests and biological samples during a day-long session at the clinic, and be willing to repeat this assessment in 2 years. The main aims are to discover the genetic and life course influences on the development of Alzheimer’s pathology, neurodegeneration and cognitive decline; explore the earliest biomarker and imaging changes in Alzheimer’s disease; validate sensitive measures of disease progression; and use these data to optimise trial design for clinical trials aiming to prevent Alzheimer’s disease. An Insight 46 protocol paper is forthcoming.

### Study member engagement and participation

To what extent cohort studies need to have high participation rates or be representative of the general population is a matter of much debate and depends on the purpose of the study [71, 72]. The NSHD study team uses various strategies to maintain its high participation rate as this helps to minimise bias [73, 74], and supports descriptive epidemiology to inform evidence-based healthcare planning [75].While attrition is least amongst more advantaged socioeconomic groups and those with higher cognitive function [40], we still maintain a sufficiently high proportion of those from more deprived backgrounds or with lower function to test many life course hypotheses.

In the last 10 years, we have asked more of study members: decreasing the time between data collections which have consisted of both postal questionnaires and visits, including travel to clinics, and more clinical assessments and biological samples. This posed a risk to participation, especially as other studies have shown that permanent withdrawal and non-participation become more common with advancing age [76], and in general participation is falling in many epidemiological studies [77]. The study team have extended the ways in which they try to maintain participation rates of at least 80%: by being ever more diligent in responding to individual queries and comments, ensuring the experience of taking part is as smooth as possible and providing ever more tailored information and feedback; and by creating an even greater sense of identity with the study by requesting study members’ personal experiences of being in the study, providing opportunities to meet other participants and the team through events held to mark the 65th and 70th birthdays, and working with them to increase the study’s public profile. The stories of NSHD study members can be listened to on our website (http://nshd.mrc.ac.uk/70thbirthday/birthdayevents/afternoon-talks/).

**Figure:**
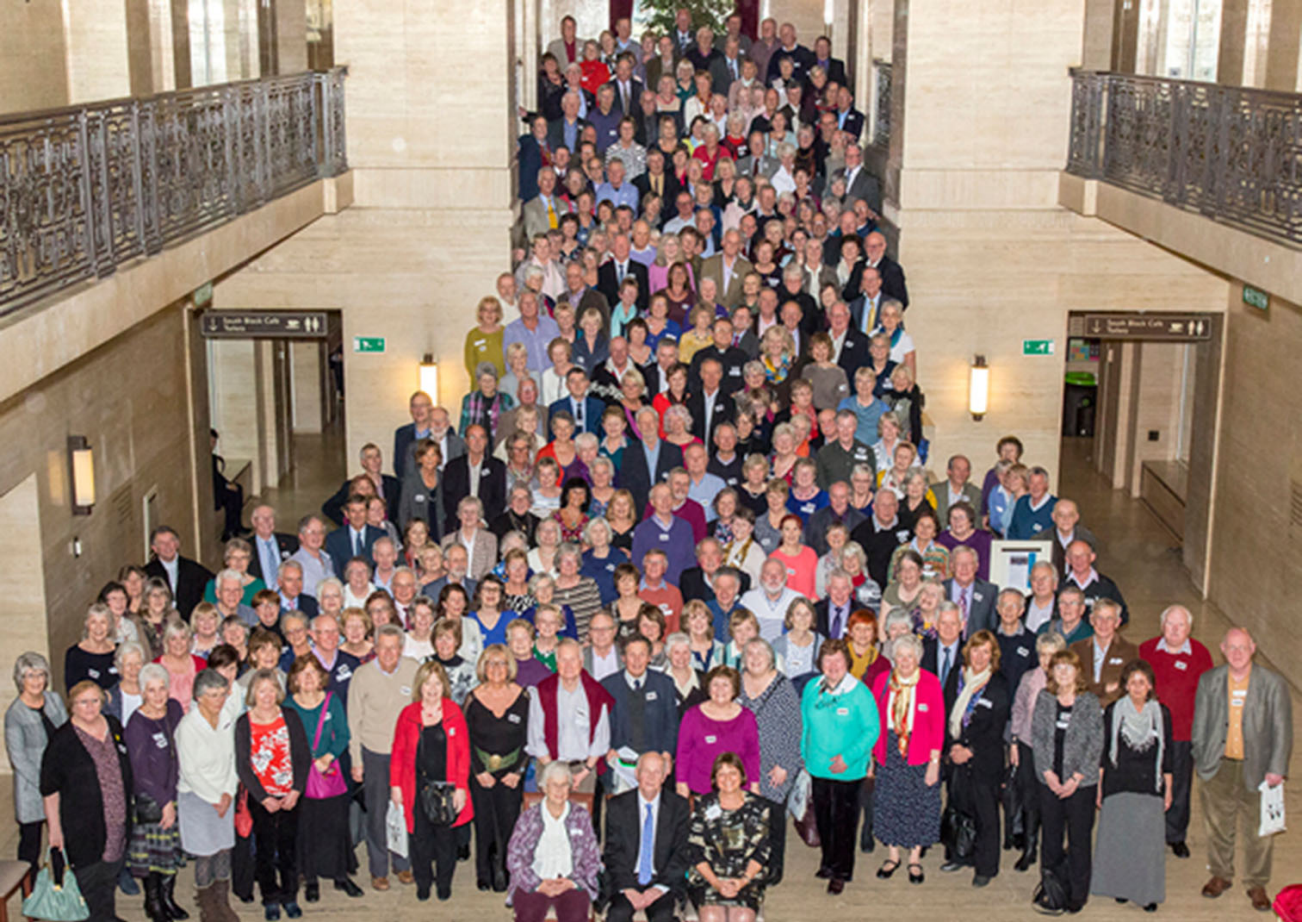
The NSHD 70th birthday party in London, March 2016

**Figure:**
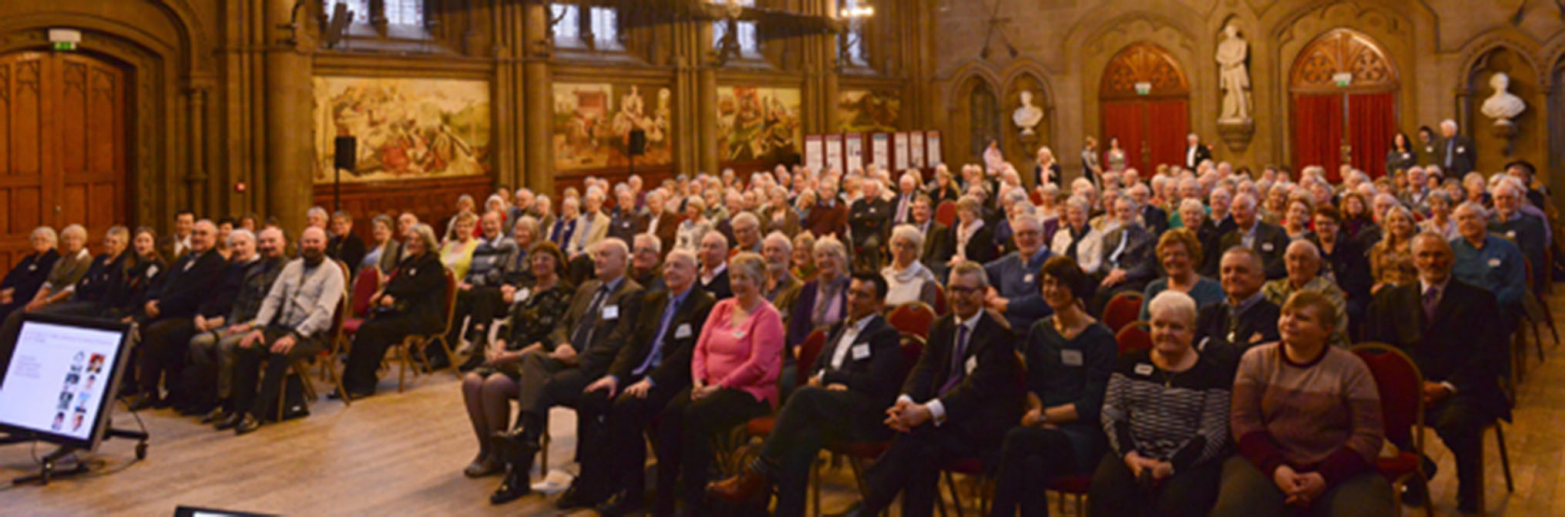
The NSHD 70th birthday party in Manchester, March 2016

Despite our concerns, the overall participation rate reached 94% at the latest data collection, and we now have some evidence that these extra efforts increased participation, even allowing for the history of past contact (Kuh et al. this issue). We will use this evidence to design the best ways to continue high participation into the 8th decade.

The neuroscience sub-study is the most demanding request we have ever made to study members. The Insight 46 team works closely with participants to fully explain the undertaking and the participants’ willingness to be involved is testament to this type of approach; to date over 250 study members have taken part in this sub-study.

## Current and future challenges for NSHD and life course epidemiology

### Developing NSHD’s scientific niche

All prospective cohort studies must find and continually evolve their scientific niche to be successful. The NSHD has survived for 70 years through an ever changing research landscape. Now, in the world of biobanks and very large cohort studies, the rise of data intensive biology, data linkage, and the promise of precision medicine [78, 79], NSHD’s scientific niche needs to be re-evaluated and re-affirmed, at a time when MRC, ESRC and Wellcome Trust are carrying out their own reviews of longitudinal resources ahead of a major shift in the structure of the UK research councils following a review by Sir Paul Nurse for the UK government on how these research councils could support UK science most effectively [80]. In this context, the team will need to show how innovative science that integrates big data with the rich prospective data archives from NSHD and other cohort studies adds scientific value. The NSHD and other life course studies with repeat measures in childhood and adult life, will continue to complement large new cohorts such as UK Biobank for many years. They can investigate how changing social and biological exposures across childhood and young adulthood, as well as during adult life, influence level and change in health outcomes, and whether later life factors are modified by those in early life. So far, output from UK Biobank has replicated some of the links between development and ageing first demonstrated in the smaller studies, using retrospective data albeit on a much larger sample (for example see [81, 82]) Over time, as novel or more finely grained adult phenotypic characteristics are discovered in these very large cohorts to be risk factors for subsequent disease, life course studies will be able to identify their early drivers.

Biomedical scientific knowledge is growing rapidly and can support a strong case for continuing NSHD until the expected 300 participants become centenarians, even in the more austere world that cohort studies are now operating in. The future scientific vision for NSHD in its 8th decade will be for the next Director to develop with the team.

### Developing dynamic concepts of health and ageing, redefining disease

The life course research undertaken on NSHD and other cohort studies, and the concepts and ideas of life course epidemiology have played their part in helping to widen the study of health and ageing beyond a traditional epidemiological focus on specific age-related diseases. The value of studying lifelong health through measures of function, capturing how function changes with age across life, and its lifetime drivers and consequences, is now widely acknowledged. While the UK came later than many other countries, such as the US, in recognising mobility and cognition as hallmarks of ageing, the wealth of birth cohort data in this country has meant we have been able to add a life course epidemiological perspective to this research area.

A broad, interdisciplinary research effort is underway investigating biomarkers of ageing both for aetiological insights of underlying biological pathways, and to test whether quantitative biomarker profiles predict age-related functional change or disease development. A current challenge for epidemiologists and cohort investigators is to work with biologists and other scientists to exploit these intensive data at the cellular and molecular level in ways that will help to redefine disease, identify common ageing mechanisms, and offer opportunities for precision medicine [83]. Rapidly developing technologies allow these data to be collected on large samples and, over time, high throughput allows costs to diminish. A challenge is to apply a life course perspective in this world of ‘omics and geroscience, to the hallmarks of ageing at the body systems [84], and molecular levels [85]; thus increasing scientific knowledge of the mechanisms underlying the developmental and early environmental origins of ageing. Life course epidemiology also needs to remain true to its roots by finding novel ways to incorporate social and cultural change across cohorts with big data on biology and the exposome. Reports on precision medicine allude to the importance of socioeconomic factors ([83] p. 43–44), but interest in social epidemiology, social medicine, and social inequalities has dropped down the research as well as the political agenda. As Brayne argues in a recent editorial, long running cohort studies should be wary of ‘attaching to the coat tails of the latest cutting edge cellular, molecular and imaging technologies,’ and maintain their wider perspective on the major challenges of human society [86].

Many years ago, Dubos recognised that health and disease reflect the ability of an organism to adapt to environmental challenges [87]. New biological and technical developments provide opportunities to study physiological resilience in more depth, in addition to the studies of psychological and social resilience which are becoming more prevalent at older ages [69]. Here, concepts and methods for studying resilience from a life course perspective need to be further developed and tested. There is still much to explore about the extent of continuity in resilience across life, or whether there are later health costs of early resilience. Increasing research on how individuals respond to environmental challenges will promote a more dynamic concept of general health based on physiological, psychological and social adaptations [88]. It encourages the collection of dynamic assessments of health and function (e.g. changes in mood, blood pressure variability), and the long-term impact of individual variability or acute events (such as acute illnesses).

### Capacity building

The deepening and widening cohort data archives through the addition of big data and record linkage, and the increase in data sharing and cross cohort collaborations is shifting the balance between primary and secondary data users in favour of the latter. These changes make it harder for early career scientists to make their mark, and be recognised for their contribution [89]; and it means that many may never be involved in collecting primary data. Training in population sciences needs to ensure that the next generation develop into innovative researchers who can think creatively, conceptually and rigorously about complex problems [90], assess the quality of data for themselves, know when (and when not) to apply increasingly complex statistical models, and how to collaborate effectively. Study teams running long-term cohorts need to offer opportunities for career development to attract some of the most able to be the next generation of primary investigators.

### Does life course epidemiology matter?

The life course approach has been incorporated into many policy documents about lifelong health and ageing (see examples in Box 1). In that sense, the field matters, although as I have discussed elsewhere [91], there are substantial challenges of translating research findings from observational studies, and life course studies in particular, into practice or policy-relevant messages for healthy ageing or for other health outcomes. Scientists, including myself, using data from NSHD and other life course studies have been primarily concerned with knowledge creation and dissemination [92], albeit including the policy implications of the findings. As researchers, we face a tension between being cautious in extrapolating our findings, and the growing demands of research funders and policy makers to demonstrate impact. Life course epidemiology will contribute to and benefit from the wider debate within epidemiology about study designs and analytical strategies for going beyond associations between exposures and outcomes to explanations based on a pluralistic approach to causal inference [93, 94]. This should strengthen recommendations for policy and interventions by ensuring they are based on the best evidence.

**Box 1:** Examples of the life course perspective in recent policy documents on lifelong health and ageing

“The evidence base for the life course approach is strong. What happens early in life (indeed in fetal life) affects health and wellbeing in later life” [96] The… diversity in the capacities and health needs of older people is not random, but rooted in events throughout the life course that can often be modified, underscoring the importance of a life course approach” [97] “Fostering healthy ageing will require a much better understanding of common trajectories of intrinsic capacity and functional ability, their determinants and the effectiveness of interventions to modify them” [97] “Transforming services for older people requires a fundamental shift towards care that is co-ordinated around the full range of an individual’s needs (rather than care based around single diseases)” [98] “Successful policy responses in an ageing population are likely to be those which take a whole life course approach” [99]	

The impact of the findings from cohort studies such as NSHD and from life course epidemiology has often been indirect through their influence on popular thinking. I agree with Keyes [95] who suggests that life course epidemiology has broadened our understanding of factors that underlie health and illness, and is part of the epidemiology of consequence that focuses on ‘what matters most for population health’ which include the ‘early life, upstream, and macro policy-related factors that are the critical drivers of many adult outcomes’ (p. 8). However, the implications from life course research, for example, about the long-term effects of early adversity for population ageing may not be attractive in the current political climate [1].

## Conclusions

Just as an individual must adapt to ever changing environmental challenges for a long and healthy life, researchers responsible for a birth cohort study must adapt to ever changing scientific knowledge and research environments. The study team must engage participants and address questions of scientific and policy relevance at each life stage to have a chance of becoming a cradle to grave study. It has been my privilege to serve the NSHD for 30 years, and to have led the study and the study team for 10 years. I acknowledge the strong team I have worked with and thank the participants who, being just 7 years older, have shown me at each stage of adult life what might lie ahead in my own life trajectory. Findings from this study were originally used to help design services for pregnant women, mothers and young children when the National Health Service (NHS) was established in 1948. Findings from this study are increasingly being used to improve services for older people at a critical time in the history of the NHS and social care.
